# Identifying the context, mechanisms and outcomes underlying collective leadership in teams: building a realist programme theory

**DOI:** 10.1186/s12913-020-05129-1

**Published:** 2020-03-30

**Authors:** Aoife De Brún, Eilish McAuliffe

**Affiliations:** University College Dublin Centre for Interdisciplinary Research, Teaching and Innovation in Health Systems (UCD IRIS), School of Nursing, Midwifery & Health Systems, Health Sciences Centre, University College Dublin, Dublin 4, Ireland

**Keywords:** Collective leadership, Realist synthesis, Programme theory, Healthcare, Teams

## Abstract

**Background:**

There is accumulating evidence for the value of collective and shared approaches to leadership. However, relatively little research has explored collective leadership in healthcare and thus, there is a lack understanding of the mechanisms that promote or inhibit the practice of collective leadership in healthcare teams. This study describes the development of an initial programme theory (IPT) to provide insight into the mechanisms underpinning the enactment of collective leadership.

**Methods:**

This IPT was informed by a multiple-method data collection process. The first stage involved a realist synthesis of the literature on collective leadership interventions in healthcare settings (*n* = 21 studies). Next, we presented initial findings to receive feedback from a realist research peer support group. Interviews with members of teams identified as working collectively (*n* = 23) were then conducted and finally, we consulted with an expert panel (*n* = 5). Context-mechanism-outcome configurations (CMOCs) were extrapolated to build and iteratively refine the programme theory and finalise it for testing.

**Results:**

Twelve CMOCs were extrapolated from these data to form the initial programme theory and seven were prioritised by the expert panel for focused testing. Contextual conditions that emerged included team training on-site, use of collaborative/co-design strategies, dedicated time for team reflection on performance, organisational and senior management support, inclusive communication and decision-making processes and strong supportive interpersonal relationships within teams. Mechanisms reported include motivation, empowerment, role clarity, feeling supported and valued and psychological safety which led to outcomes including improvements in quality and safety, staff and patient satisfaction, enhanced team working, and greater willingness to share and adopt leadership roles and responsibilities.

**Conclusions:**

This study has identified preliminary support for the contexts, mechanisms and outcomes underpinning the practice of collective leadership. However, it must be noted that while they may appear linear in presentation, in reality they are independent and interlinked and generative of additional configurations. This paper contributes to the nascent literature through addressing an identified gap in knowledge by penetrating below the surface level inputs and outputs of an intervention to understand why it works or doesn’t work, and for whom it may work.

## Background

Effective leadership in healthcare settings has long been acknowledged as a driver of quality care delivery, work engagement and achievement of performance targets [1]. In a recent review of the evidence for leadership in health settings, it was concluded that leadership is the most influential factor in determining organisational culture and is crucial for health services improvement [2]. In healthcare contexts, a traditional approach to leadership is prevalent, where the focus is on the individual as leader, and that person leads and is accountable for the work of the team. However, leadership with a strong emphasis on hierarchy can inhibit a positive safety climate due to fear of blame and repercussions for voicing concerns [3] and can potentially give rise to bullying and intimidation of more junior colleagues [4]. Moreover, healthcare is increasingly delivered by multidisciplinary teams, where medical staff, nursing staff, health and social care professionals, and other professional groups are expected to collaborate and contribute their expertise to deliver optimal care to the patient. Thus, the traditional hierarchical approach to leadership is no longer appropriate in the current healthcare environment [5, 6] as leadership is increasingly considered a skillset that should not be limited to senior managers in formal positions, but something to be embraced by staff at all levels [7].

This paradigm shift reflects a move in focus from the individual as leader to the emergent and informal leadership evident within a team or group [8]. Collective leadership and other plural approaches to leadership (e.g., shared or distributed leadership) have been associated with enhanced team effectiveness and team performance outcomes [9, 10]. Such approaches emphasise the relational aspects of leadership. Implicit in this is the acknowledgement that leadership is not necessarily the responsibility of, or located in, one individual, but leadership may be considered as a property of a team or work group. In such instances, there are inclusive and shared approaches to responsibility and accountability for the team’s performance and operations. This approach to leadership may be defined as a dynamic team phenomenon, where the interaction of team members lead the team by sharing leadership roles and responsibilities [9, 11], with individuals adopting leadership roles where they have the expertise and motivation to do so [12]. Whilst there is accumulating evidence for the effectiveness of collective leadership in healthcare [13], that is, the outcomes of collective leadership, there is a lack of understanding about how collective leadership interventions operate (reactions and reasonings of actors, i.e., mechanisms), and the conditions which promote or inhibit these outcomes (contextual conditions) [14]. The focus of this study is the context-mechanisms-outcome configurations observed that enable/inhibit the outcomes observed when collective leadership is in practice. Scholars have identified the role of context as an important avenue of study [9] and the “conditions under which particular aspects of team leadership affect specific mechanisms” at the team, unit, system and organizational level of analysis [15]. This study adopts a realist methods approach to provide insight into the mechanisms that are triggered or inhibited by collective leadership in specific contexts to elucidate how certain outcomes (such as improvements in quality and safety, effective teamworking and staff satisfaction) are achieved.

Realist evaluation is a theory-driven approach to research emanating from scientific realism. Realist approaches offer a means of conducting applied evaluation that recognises that research is being conducted in complex, open systems and considers the significant role of pre-existing social contexts in implementation and evaluation [16, 17]. Realist methods understand social programmes as social systems, characterised by the interplay of micro and macro social processes, and of structure and agency [17]. Pawson and Tilly asserted that there was a need to understand more than intervention effectiveness alone and argued that in order for evaluations to be useful, it was crucial to examine ‘what works for whom, in what context, to what extent, how and why’ [17]. Programmes are theories incarnate [18] and realist evaluation appreciates that interventions may operate in different ways for people in different contexts. Thus, realist evaluation is a logic of inquiry that goes further than merely exploring the surface level inputs and outputs of an intervention, by discerning the psychosocial mechanisms (M), that is, the internal reactions and reasonings, that trigger intervention outcomes (O) in specific contexts (C) of implementation [14]. Mechanisms have been defined as the unobservable implicit processes that occur in individuals’ minds due to the intervention; they elucidate what it is about a programme that makes it work [16, 17, 19].

The realist approach demands that the relationship between the context, mechanisms and outcomes in an implementation setting be explored. The context-mechanism-outcome configurations (‘CMOCs’; i.e., C + M = O) that are uncovered become part of an explanatory theory (the initial programme theory; IPT) to be tested and refined. The function of the IPT is to describe and explain insofar as possible how and why the programme (i.e., the intervention) may be working for some people and not others, depending on which mechanisms are or are not triggered in specific contexts. These chains of inference enable the exploration of generative causation, by explicitly linking the triggering of mechanisms to contextual conditions and specific outcomes. Through elicitation of the patterns of CMOCs that are evident across settings (‘demi-regularities’), one can establish the CMOCs that operate as the common thread of an intervention across various contexts. The evaluation of an intervention then should test and refine these theories and hypotheses.

To our knowledge, this is the first study to adopt realist approaches to explore why and how collective leadership interventions operate to trigger mechanisms that lead to certain outcomes. This study builds an explanatory theory to interrogate the key contextual conditions, mechanisms and outcomes and their interactions to provide insight into how collective leadership can be effectively implemented to lead to desired outcomes, including practice of collective leadership, improved team working, and improvements in quality and safety culture [14].

## Methods

### Design: programme theory development

The development of the programme theory to explicate how collective leadership interventions operate to produce outcomes will be guided by the realist evaluation cycle approach [17] previously outlined in the published protocol paper describing this research [14]. The four stages of the research to inform and finalise the IPT involved a realist synthesis of the literature on collective leadership interventions in healthcare; presentation and feedback on initial findings to a realist research peer support group; interviews with members of teams who have been identified as successfully working collectively in the healthcare system; and expert panel input to refine and finalise the IPT (see Table 1). This paper reports on the development of the programme theory to explore implementation of collective leadership interventions in healthcare: what works, for whom, why, to what extent, and in what circumstances? The methods and results are described in accordance with RAMESES II guidelines on the reporting of realist evaluation research [18].

**Table 1:** Summary of steps to develop and refine IPT

Stages of consultations	Source of expertise	Date
Early iteration of IPT following realist synthesis presented	Realist methods peer support group	March 2018
Discussion of IPT	Research team and programme developers	April 2018
Refinement following analysis of interview data	Key informants (interviewees)	May–June 2018
Refinement of IPT	Research team and programme developers	July–September 2018
Input from expert panel; prioritisation of CMOCs for testing	Programme developers; experts in collective leadership	November – December 2018
IPT finalised for testing	Programme developers	January 2019

#### Realist synthesis of the extant literature

A realist synthesis of the literature was conducted on studies retrieved during a systematic review of interventions to develop collective leadership in healthcare settings (full search strategy available in published paper) [13]. Twenty-one papers were assessed for rigour and ability to add to the developing programme theory and 19 were included in the final analysis (two were excluded due to insufficient information to inform IPT). Informed by previous research [20, 21], an extrapolation template was designed to identify contextual conditions that enabled or inhibited mechanisms or psychosocial drivers for collective leadership in practice and related intervention outcomes. Context-mechanism-outcome configurations (CMOCs) were extrapolated from each paper and demi-regularities (patterns across studies) were identified to inform the first iteration of the IPT. This initial IPT was presented to a realist research peer support group for feedback and advice.

#### Interviews with individuals on teams working collectively

Senior leaders in the healthcare system assisted the researchers in identifying effective healthcare teams in the healthcare systems. We asked managers to identify those teams with a flattened hierarchy and where collective leadership was evident within the teams. Individuals from these teams were provided with an overview of the research and were invited to take part in a one-on-one interview with a researcher to explore their experiences of working within the team, team processes, why and how the team was working collectively, and the impact on team working and safety culture (paper in preparation). Twenty-three individuals from three teams took part in interviews. Informed consent was sought from all participants in advance of being interviewed. Participants were from a range of backgrounds, working in various roles within the health system. Table 2 summarises the characteristics of interviewees. Interviews were audio-recorded and transcribed verbatim and NVivo11 was used to manage and analyse the data [22]. Data analysis employed a retroductive approach using both inductive and deductive logic to interrogate the causal factors that may have operated to produce outcomes [23]. Interview data was first deductively analysed to support and refute CMOCs that had been extracted from the literature and an inductive analytical approach also enabled the extrapolation of new CMOCs. The approach aligns with the methodological approach elaborated in detail elsewhere [24]. Briefly, NVivo enabled the creation of nodes (codes) and memos (to document reflections and ideas) relevant to each data source and nodes were created for each hypothesised CMOC and new child nodes were added if new contextual conditions, mechanisms or outcomes were identified. The use of NVivo to support the analysis enabled transparency in the process through the tracking of the iterative refinement of the IPT. The first author conducted this analysis and 20% of the data were double coded by the second author to ensure confidence in the findings. Through discussion, the co-authors refined and finalised the CMOCs prior to presentation to the expert panel.

**Table 2:** Sample characteristics of interviewees from effective teams

Participant number	Role	Time on team
**Team 1**		
F01	Head of Clinical Services and Business Planning	2 years
F02	Pharmacist	12 months
F03	Occupational Therapy Clinical Specialist	18 months
F04	Occupational Therapy staff	18 months
F05	Medical social worker	15 months
F06	Occupational Therapy staff	10 months
F07	Senior Physiotherapist	15 months
F08	Occupational Therapy staff	18 months
F09	Physiotherapist	1 month
F10	Senior Physiotherapist	2 months
F11	Dietician	18 months
F12	Speech and Language Therapist	18 months
**Team 2**		
C01	Consultant	4 years
C02	Senior Mental Health Social Worker	6 years
C03	Senior Social Worker	11 years
C04	Clinical Psychologist	9 months
C05	Social Care Leader	17 years
C06	Occupational Therapist	10 years
**Team 3**		
P01	Research Coordinator	3.5 years
P02	Research Scientist	12 years
P03	Consultant	7 years
P04	Clinical Psychologist	6 years
P05	Project Manager	7 years

#### Expert panel input

Programme designers and experts in the fields of team working, collective leadership and safety culture (*n* = 5) provided individual feedback to the research team to inform and refine the IPT. Further details on the expertise of the panel members is included in Appendix. The 12 CMOCs extrapolated from the previous stages of development were presented and feedback sought regarding prioritisation of CMOCs for testing. Following this consultation, minor amendments were made and no additional potential CMOCs were identified. The panel also engaged in a ranking exercise to establish the relative importance of CMOCs to the implementation (or not) of collective leadership. As a result of this, seven CMOCs were prioritised by the expert panel and are presented in detail in this paper as the programme theory.

## Results

**Fig. 1:**
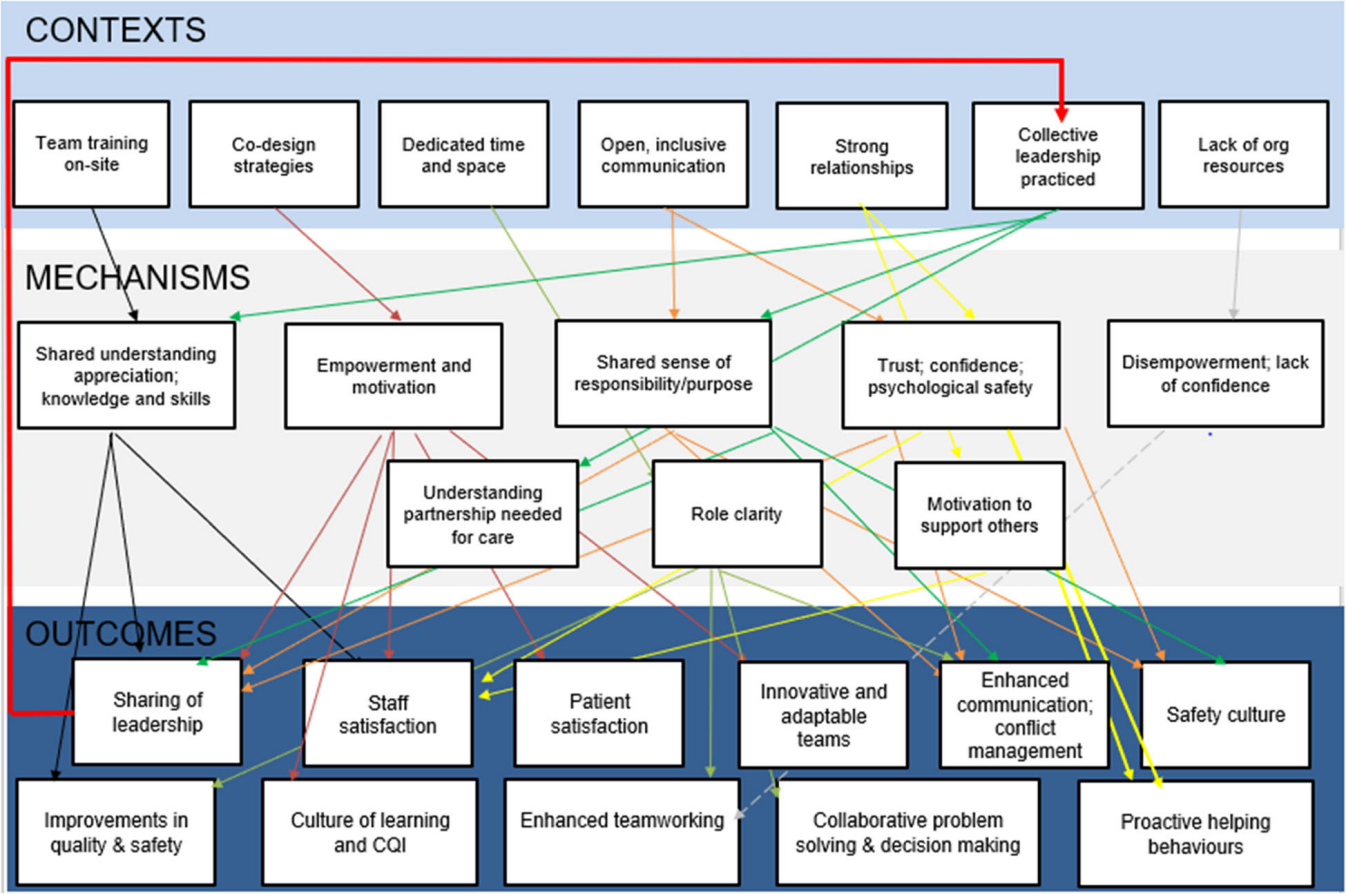
Initial Programme Theory for collective leadership, depicting context-mechanism-outcome configuration

This section describes the results following the iterative process to develop and refine the IPT. The seven CMOCs prioritised for further testing are considered in detail below, with accompanying evidence for each derived from one or more of the development stages (see Table 3). We also present Fig. 1 which more accurately depicts the complexity of the relationships between contexts, mechanisms and outcome. It is important to note that whilst the CMOCs are often presented as ostensibly linear relationships, the reality is much more complex, as many of the CMOCs are interdependent and interacting. The CMOCs extrapolated and presented here are the major relational patterns (demi-regularities) that have been observed across multiple contexts. We will elaborate on this further in the discussion of findings.

**Table 3:** Initial Programme Theory and supporting evidence

CMOC	Context	+ Mechanism	= Outcome	Evidence
1	Team training on-site	• Shared understanding and appreciation of others • Confidence in enhanced knowledge and skills in collective leadership	• Greater staff satisfaction through enhanced interdisciplinary teamworking • Improvements in quality and safety • Enactment of shared leadership behaviours	[25–31]
2	Team given permission/encouragement to self-manage and use co-design or collaborative approaches for improvement	• Empowerment and motivation through sense of shared responsibility for team performance	• Teams more innovative and adaptable, characterised by a culture of learning, collaboration and continuous quality improvement • Staff satisfaction • Patient satisfaction • Adoption and sharing of leadership roles and responsibilities	[26, 32–36] Interview data
3	Dedicated time to reflect on and discuss team operations	• Greater role clarity	• System improvements, such as improvements/greater efficiencies in team processes around patient care • Enhanced teamworking; increased productivity • Effective team communication • Greater involvement of frontline staff in decision-making	[26, 27, 32, 33, 37] Interview data
4	Open, regular and inclusive communication and decision-making processes	• Enhanced trust and psychological safety • Sense of shared responsibility	• Effective communication, knowledge sharing and conflict management • Safety culture characterised by greater safety awareness and open discussion of issues • Team leaders willing to share leadership responsibilities and adoption of leadership responsibilities by team members	[26, 28, 31, 33, 38, 39] Interview data
5	Lack of organizational support/resources, senior clinical support, or a strong hierarchical culture	• Disempowerment • Lack of confidence in approach	• Avoidance of team working	[28, 32, 40]
6	Strong, supportive interpersonal relationships (formal and informal)	• Motivation to support others due to shared burden/responsibility • Trust and confidence in others’ expertise	• Enactment of proactive helping behaviours (role blurring) that enhance team performance • Staff satisfaction and retention	Interview data
7	Collective leadership is practiced	• Understanding that partnership needed for effective patient care • Internalization of collective leadership concepts; shared sense of responsibility for team • Recognition and understanding of skills and expertise of others	• Patient satisfaction • Improvements in patient safety and care quality • Willingness to speak up • Senior colleagues more open and accessible • Inclusive and collaborative team working characterised by a ‘give and take’ approach	[25, 28, 30, 31, 35, 36, 39, 40]

In the ‘Evidence’ column, ‘Interview data’ refers to evidence from the interview data we collected for this study, where a CMOC was evident across at least two of the three teams. The numbers relate to supporting references from the published literature

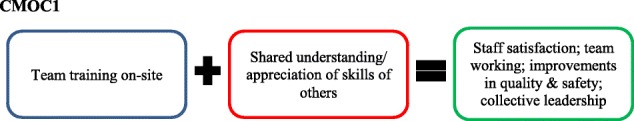



One of the most powerful and frequently observed contextual enablers of intervention success was contexts in which multiple professionals received training together as a team and where training was conducted on-site with teams. Often, additional resources were required to enable successful implementation of collective leadership in the setting, such as internal or external coaching, organisational support to ensure teams are released and supported to attend training together, as well as renumeration and meals when training was delivered over an extended period.

“The greatest success was bringing together physicians and nurses to lead in ways that reinforced that patient care is truly about partnership.” (p. 26) [25]

In such contexts, seven studies suggested that the mechanism triggered was the development a shared understanding among the team that partnership among various professions on the team is required for effective and high-quality patient care.

With this shared understanding, contexts where training occurred together as a team was purported to break down the ‘silo mentality’ (a narrow focus on one’s own profession or work unit to the extent that there is little consideration of the views of those outside the profession or unit) that may exist among professions on a team and encourage a shift in mindset toward collective rather than individual achievement. Once this silo mentality was challenged, team members had a greater understanding and appreciation of each other’s roles on the team. This led to positive outcomes for staff, patients and the organisation. In such contexts, staff job satisfaction was enhanced through more effective interdisciplinary teamworking and collaboration, with reported improvements in patient safety and quality indicators. There was a perceived flattening of the hierarchy in the team, with increased staff engagement and enactment of collective leadership behaviours reported at all levels (from both formal and informal leaders).

“‘We used to be in silos. Not a lot got done. No one took ownership for progressing things. Now we are getting things done, there is delegation, we have responsibilities within the team’” [26]

“The response to the programme was highly positive; engagement exceeded our expectations. Most survey respondents reported improved willingness to take on a leadership role within their team (93 per cent)... The “in-house” training model promotes development of social capital across different disciplines and levels of management. This overcomes the organisational barrier of inter-professional tension commonly recognised as hindering leadership development ” [27]

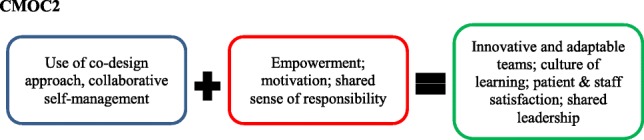

Collective leadership introduced to contexts where teams are involved in, and responsible for, improving staff experience, patient care services and/or teamworking through collaborative or co-design approaches (and where staff are resourced with time and space for reflection and discussion), fosters a sense of shared accountability and responsibility for the team to enhance patient care. Team members become more interdependent as the team becomes more autonomous in improvement efforts. Contexts characterised by collaborative and co-design approaches empower staff and can trigger enhanced motivation and a commitment to improvement. One study observed “a change in the way people thought about care rather than just a change in the process” [32].

“A crucial part of the new team-based approach has been the greater distribution of leadership responsibilities to the teams, providing authority to those “individuals who are willing to engage in change efforts” (Fitzgerald et al., 2013).” [33]

“We were told by senior management at the beginning that we could do whatever we felt was necessary to improve the outcome for patients – quality and safety – and I think that was really important that we were supported in that.” (Interview, Team 1, F04)

Outcomes observed as a result included teams became more effective, innovative and adaptable, and a culture of learning and continuous quality improvement was embedded when there was a sense of a shared responsibility for the team’s performance. This in turn was associated with improved staff satisfaction, the adoption and sharing of leadership roles and patient satisfaction with care services.

“Another benefit is the increase in personal accountability evident in staff behaviours. No longer are problems and issues left for management to ‘fix’ … The turnover rate was the most dramatic change that the department experienced as a result of engaging staff using shared leadership principles. The rate has decreased from 40% in 2001 to 4% in 2004.” [34]

“We’re always striving to be better and to do better.” (Interviews, Team 1, F06)

“Participants reported a new way of working with patients, families and staff as co-producers of the service.” [32]

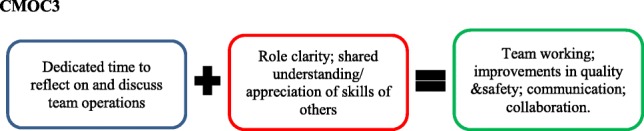

Contexts in which teams were supported by organisations with dedicated time and space to reflect on their practice and their team operations, including development of patient pathways and team processes, was highlighted in several papers as an important contextual enabler that facilitated intervention success.

“The months of preparation gave members of the department an opportunity to talk at length about the new approach, decide on how the teams would work together, and experiment with changes to systems as to the timing of appointments, and methods of arranging follow up appointments. The experiments then enabled the department, as a whole team, to decide which approaches seemed best.” [33]

Positive outcomes were triggered through fostering role clarity among team members. When this mechanism was triggered, system changes and improvements such as reduction in waiting lists, enhanced teamworking and “an increase in productivity” [33] were observed. There was more effective communication reported between team members and broader team involvement in decision-making.

“[The intervention] gave the teams the opportunity to meet together as a cross-disciplinary team with dedicated time out, a facilitator to work with them and an expectation that they would set goals and work towards them. This was a new experience for groups. As a mechanism of change, this bringing together of a specialised team and the provision of facilitated time out for them could be seen as a powerful model for improvement in the NHS [National Health Service], particularly in those areas where multi-professional teams need to co-operate. It creates space and combines this space with the provision of operational tools and techniques. This seems to be a powerful combination and to be effective in contributing to changes in practices and procedures.” [26]

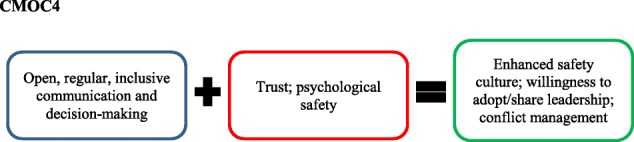

In implementation settings where there is open, regular communication and team members have the opportunity to engage in inclusive decision-making processes to encourage contribution and collaboration, teams develop trust between members, enhance psychological safety within the team and foster a sense of shared responsibility. This may occur through structured processes to enable input from all team members, such as team safety huddles or multi-disciplinary team meetings, or can occur in settings characterised by a “fundamental orientation towards inclusiveness” [38]. One example from the literature describes the benefit of team huddles as a feature of the context that enabled effective communication and knowledge sharing.

“When this reciprocity and mutual influence is acknowledged and formalized, it can become an institutional feature of work (Gronn 2002), such as a new model of care. An example is the use made of huddles by Team A, to harness every member’s input when a situation is analyzed, for which a plan needs to be developed or a change to care needs to be made” [28]

When trust and psychological safety are activated through inclusive communication and mutual influence in decision-making, this results in the emergence of a culture of learning in teams, where team members have enhanced safety awareness and feel comfortable openly discussing patient safety issues and concerns.

“The hierarchical structures of hospitals are hard to break down and I think we are further along the way to get to a place where people feel there is a level playing field for everybody.” (Interview, Team 3, P03)

“I think it is that no blame culture. It is being able to actively reflect on something rather than ‘Why didn’t you?’ or ‘You should have’ – dialogue is quite different, it is: ‘Lets learn from this and move on.’” (Interview, Team 1, F08)

“You are not afraid to ask and that is a very good culture to have”. (Interview, Team 3, P01)

Once trust and psychological safety was fostered, it resulted in team members adopting more leadership responsibilities and with team leaders more open to input from team members.

“Senior physiotherapists discussed patients more with junior physiotherapists; team members would look at referrals together before they went into a new patient clinic, and talk about the assessment beforehand … In other words, team members took on more leadership responsibilities” [33]

“Team leaders were more willing to listen to others, to take on board ideas put forward by those from other professional backgrounds, and to relinquish some control.” [26]

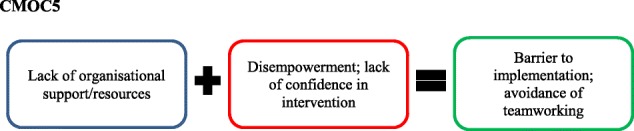

In contexts where there is a lack of organizational support/resources, senior clinical support/engagement, or a strong hierarchical culture, when collective leadership is introduced, mechanisms triggered serve to operate as a barrier to change. Mechanisms include feelings of disempowerment and a lack of confidence in the approach. Such contexts prevent the internalisation of collective leadership concepts, result in a resistance to teamworking and reduce the likelihood of successful implementation.

“Some participants felt they lacked the necessary support and appropriate climate to implement new ways of working. Although the programme recognised the importance of distributed leadership, it may be that this is difficult to achieve in organisations that are hierarchical in structure.” [32]

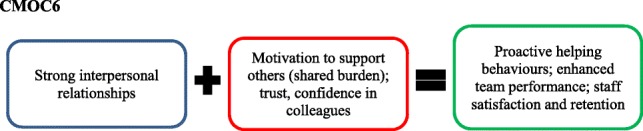

Across the three teams interviewed, strong, supportive interpersonal relationship were explicitly linked to a motivation to support others and share the burden of the team’s work. This team-based approach to workload management encouraged the enactment of proactive helping behaviours where team members would explicitly offer each other support if they perceived someone to be managing an excessive workload or having a bad day. In such instances, participants described how others would “rally around” (Interview, Team 2, C03) those who may have need help and described a process of “give and take” (Interview, Team 2, C01; Team 1, F06), where team members were happy “venturing into each other’s spheres” (Interview, Team 1, F07) to provide support and assistance. This supportive work culture was asserted to enhance team performance.

“I think it is that willingness to help each other out. It is not just seen as my role or your role. I think it is kind of that we are all there to help the patients” (Interview, Team 1, F06).

Trust and confidence in each other’s expertise and skills were also enabled through the development of strong and supportive interpersonal relationships. Participants stated that development of close working relationships over time was directly linked to their confidence in the clinical judgement of others. It was often mentioned that team members attended informal outings together and regularly celebrated each other’s successes and personal milestones. This resulted in greater staff satisfaction and retention, as team members felt supported and comfortable with their colleagues.

“I suppose the months and years you spend together you just kind of trust people’s kind of, like, clinical judgement really well.” (Interview, Team 1, F12)

“[Its] the sense that I’m never completely on my own with something that is causing me anxiety and that’s because of the interpersonal relationships … I don’t want to go anywhere, I’m happy here. I mean that’s a sign of a good team” (Interview, Team 2, C04)”

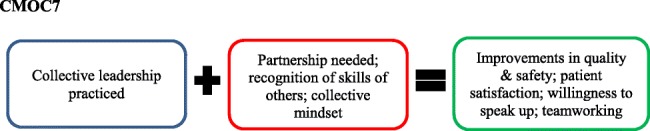

In contexts where shared/collective leadership is practiced, there was strong evidence from the literature that this enabled a shift from an individual towards a more collective mindset, whereby team members internalised the concepts of collective leadership and developed an understanding of the importance of interdisciplinary partnership for effective patient care.

“Our study generated evidence of spontaneous collaboration and a genuine shared role space existing between nurses and PCWs [personal care workers] in Team A … evidence of the close understanding between team members, leading to more sensitivity in care delivery.” [28]

This led to positive outcomes for patients, including improvements in safety and quality of care, as well as enhanced patient satisfaction with care. Positive outcomes for staff were also observed. Several studies reported that team members were more willing to speak up, perceived their team leader and senior colleagues as more open and accessible and reported a more inclusive and collaborative team working.

“Co-leadership exerted in an integrated and co-located centre allowed the managers to deal with service users’ needs and problems in a more holistic and efficient way.” [39]

“They also felt that the award had helped enable each of them to develop as leaders in their own spheres, had made the team leader more willing to listen to them, and had given them the confidence to put their own ideas forward more” [26]

An enhanced recognition of the skills and expertise of team members, and a sense of being valued, were triggered in contexts where collective or shared leadership was practiced included and for their expertise and judgement. This resulted in interdisciplinary collaboration in patient care.

“Being interested in and willing to invest time in collaboration and in learning about each other’s responsibilities and sector-specific [health and social care] activities was crucial to understanding and managing the big picture … By the advantage of being two managers with different knowledge and responsibilities, the managers could complement each other’s areas of expertise.” [39]

## Discussion

This paper describes the results of a rigorous and iterative approach to the development of an initial programme theory to evaluate the impact of a collective leadership intervention. Through realist synthesis of the extant literature on collective leadership in healthcare, interviews with individuals on teams that are leading collectively, feedback from a realist research group and expert panel input, we extracted and refined seven CMOCs that together offer an initial programme theory of how collective leadership triggers mechanisms in specific contexts that lead to patient, staff and organisational outcomes.

The impact of holding team training in collective leadership on-site was a contextual enabler that emerged strongly from the literature (CMOC1). This facilitated a shared understanding and appreciation of colleagues and confidence and skills to enact collective leadership in practice. Typically in healthcare, professions are trained in silos, with little (if any) training on how to operate as a member of a multidisciplinary team [26]. Yet, this team training is crucial for the practice of collective leadership, as a shared understanding of the skills and expertise of others in a pre-requisite for the sharing of leadership roles, which is defined by as appropriate when individuals have the relevant motivation and expertise to do so [12]. The busyness of the healthcare environment precludes prospects to develop this understanding without dedicated team training opportunities.

Additionally, in healthcare settings there can be a perception that protected time for teams to review their performance is not feasible, given the demanding environment and high workload [41]. Yet, there is strong evidence that teams that take time to reflect on their processes and objectives are more effective [42] and demonstrate better individual and organisational outcomes [43]. This team reflexivity has been associated with innovation, even in busy and demanding healthcare contexts [44]. Our research found that when teams have dedicated time to reflect on team operations, this can trigger role clarity among team members and clarity regarding the sources of various types of expertise and skills within the team (CMOC3). This reflexivity enables teams to effectively self-monitor and identify improvement targets and link those to team members, given their role or skill set [45].

In contexts where there is open, regular and inclusive communication and decision-making processes, implementation of collective leadership enhances trust and psychological safety and instils a shared sense of responsibility for the team’s performance (CMOC4). A key characteristic of an effective team is psychological safety and the willingness to speak up to senior colleagues and the enactment of inclusive and openness on the part of senior clinicians and leaders [46, 47]. When psychological safety is evident, teams are more innovative are more engaged in quality improvement work [47]. Psychological safety serves to promote a safety culture within the team, where is there greater safety awareness brought about by openness to discussion of problems and challenges, thereby facilitating communication and knowledge sharing. Building trust and psychological safety enhances the adoption of leadership roles and makes leaders more willing to share leadership.

When collective leadership was observed as an outcome, the mechanisms of empowerment and motivation were observed in contexts where teams co-designed or were given permission to self-manage their team processes and improvement efforts (CMOC2). Staff engagement is critical to organisational outcomes in healthcare. West et al. have demonstrated the profound impact of staff engagement on patient satisfaction, patient mortality, infection rates and staff absenteeism [48], underlining the value of a collective leadership approach that can engage staff and encourage them to adopt a leadership role in ensuring optimal team performance. At the group level, collective leadership interventions may foster climate of psychological empowerment climate, which may be defined as a shared perception of empowerment related to “meaningfulness, competence, self-determination and impact” [49]. The engagement fostered through co-design and collaborative efforts enables teams to become innovative and adaptable and create cultures of learning and continuous quality improvement and is promoted by cultivation of a climate of empowerment.

In contexts where teams were characterised by strong and supportive interpersonal relationships, motivation to support others was triggered due to a perceived shared burden of work (CMOC6). Strong relationships also engendered trust and confidence in the skills and expertise of others. Research demonstrates that social support is a critical antecedent to the effective sharing of leadership roles and responsibilities [50]. In instances where this social support was evident, team members were likely to engage in proactive helping behaviours and role blurring to support each other and share the workload. Houghton et al. have proposed group-level caring as a process whereby team members actively look out for the interests on one another [51]. In line with social exchange theory, they assert that the norm of reciprocity operates to encourage peers to reciprocate the behavioural investments of others and this can lead to proactive helping behaviours which can impact positively on team performance [51]. This resonates with previous research which demonstrated that shared leadership was a predictor of teamwork, altruism and helping behaviour [52]. It is not surprising, therefore, that when strong supportive relationships are evident, collective leadership interventions can generate a climate of empowerment that leads to greater staff satisfaction and staff retention.

It was strongly evident from the extant literature that a lack of organisational or senior management support for collective leadership programmes resulted to unsuccessful implementation and an avoidance of teamworking, due to the disempowerment and lack of confidence of staff in the collective leadership approach (CMOC5). This resonates with previous research which emphasises the necessity of effective management-staff relations in translating evidence into action and change [53]. Due to the strong hierarchical culture in healthcare organisations, it has previously been observed that collective approaches to leadership can appear counter-intuitive and be met with scepticism [54]. Furthermore, organisations may state their support for the practice of collective leadership, but not implement the organisational changes that may be necessary to enact its practice. For example, research has illustrated how organisational structures and professional and managerial hierarchies can constrain participants’ leadership capacity and opportunities [55]. Such contexts can produce negative effects as staff may develop a ‘learned helplessness’, where they feel powerless to improve some aspect of the work experience or environment. This may lead to a reluctance to engage and has been identified as a contributory factors to allowing poor care to continue [56].

The final CMOC extrapolated related to contexts in which collective leadership was practiced (CMOC7). This may be considered a ‘ripple’ CMOC as it emerges after a programme has been successfully implemented and illustrates the additional impacts observed when collective leadership is practiced. When collective leadership is evident, there is a recognition of the need to partner with other experts to deliver optimal patient care. In turn, this results in improved patient safety and quality of care and enhanced team working. Effective team-based working in healthcare is associated with meaningful improvements in patient mortality. One study concluded that 5% more staff working in structured well-functioning teams was associated with a 3.6% lower patient mortality rate [48]. Another key outcome observed was willingness to speak up. Given our findings, we contend that collective leadership training can be a beneficial resource to promote psychological safety, a patient safety culture and effective teamworking.

This research contributes to the emerging research around collective leadership in healthcare settings by exploring the extant literature and collecting empirical data to interrogate the mechanisms and causal factors driving outcomes in specific contexts of implementation. Whilst previous research has explored the efficacy and effectiveness of collective leadership interventions, there has been little attention on the mechanisms of action and how specific contexts may operate to trigger or inhibit mechanisms from firing. The focus on mechanisms of action is a key feature of the realist approach to explore the ‘black box’ of evaluation. Dalkin et al. [19] define mechanisms as “a combination of resources offered by a social programme under study and stakeholders reasonings in response” (p.3). These invisible reactions and reasonings drive specific programme outcomes and thus, understanding how these mechanisms are triggered (or not) in specific contexts helps explain how and why a collective leadership programme is operating to drive outcomes. The programme theory presented here offers plausible, evidence-based hypotheses of how collective leadership interventions operate to drive specific outcomes. These plausible hypotheses provide a platform for the next stage of testing to enable further refinement.

There are, inevitably, limitations to this research. Firstly, there were relatively few studies on collective leadership interventions in healthcare retrieved and this may have limited our ability to identify additional significant CMO patterns across studies. Given that this is the first study using realist methods to explore collective leadership in healthcare, this work represents a first step to support the field in furthering our collective knowledge of the contexts and mechanisms underpinning the success (or otherwise) of such interventions. Within the realist approach, theory building is an on-going process. This programme theory offers a practical guide and starting point for researchers in the field to operationalise and evaluate interventions of this nature. Future research implementing and evaluating collective leadership interventions should test the hypotheses derived to further inform and refine the programme theory towards development of a middle range theory. This middle range theory should capture the ‘core’ aspects of how the intervention operates at the individual, team and organisational levels and will be broadly generalisable to other contexts. Thus, further testing of this IPT using realist evaluation is a natural next step.

Whilst the elaboration of the CMOCs is ostensibly linear, the reality is far more complex. Rarely are CMOCs neatly discrete as presented in realist evaluation of complex phenomena. There is inevitably interaction and interdependencies between CMOCs: they influence each other by their presence or absence. The CMOCs explicated here represent the over-arching relational patterns or ‘patterns of aliveness’ [57] that emerged through the study of multiple data sources and research contexts. As advised by Braithwaite et al. [58], we should not ignore this complexity but rather embrace and document it. Despite the challenges it presents: “we must grapple with the world we actually inhabit, not the one we wish we did” [58]. Pattern-based approaches in intervention contexts can be effective to bring into focus various dimensions of complexity and enable sense-making [59] using a realist lens.

Another point it is important to acknowledge is that by presenting and testing CMOCs as quasi-linear chains, they are vulnerable to becoming self-reinforcing. It is therefore crucial for researchers not to be too prescriptive in testing and be open and receptive to new or alternative explanations. It is such an approach that will facilitate advancement in our understanding of the mechanisms underpinning collective leadership, and how different contexts will trigger or inhibit different mechanisms and outcomes at various times.

## Conclusions

While collective leadership is not a panacea to solve the wicked problems inherent in healthcare settings, and there are inevitably occasions that require a more hierarchical leadership approach [60], the evidence strongly suggests that collective leadership interventions can promote more effective teamworking, enhance quality of care and patient safety and improve staff and patient satisfaction [13]. This study has gone beyond the question of effectiveness by interrogating the causal and generative mechanisms that result in the outcomes observed when collective leadership is practiced. This paper contributes to the nascent literature through addressing an identified gap in knowledge by penetrating below the surface level inputs and outputs of an intervention to understand *why* it works or doesn’t work, and for whom it may work. It provides a starting point for researchers in the field to test these plausible hypotheses to refine and deepen our understanding of how collective leadership interventions operate. A key strength of this study was the multi-step, multiple method processes delineated, an approach which incorporates best evidence from the literature, empirical data where collective leadership is in routine practice, and methodological and subject matter expertise through use of reference panels. Future work should seek to refine and expand this IPT through further testing.

## Data Availability

Data is openly available from the published papers reviewed in this paper. Interview data are not publicly available due to their containing information that could compromise the privacy of research participants.

## Appendix

**Table 4:** Further details about expertise among members of Expert Panel

Panel Member	Background/Role	Areas of Expertise
Programme Designer 1	Psychologist, experienced health systems and services researcher, Professor of Health System	Leadership, evaluation, healthcare safety, staff engagement, healthcare management
Programme Designer 2	Speech and Language Therapist, senior hospital manager, PhD candidate	Healthcare management, transformation and change management, team working
Programme Designer 3	Psychologist, experienced healthcare researcher	Collective leadership, research evaluation, team working.
External Advisor 1	Professor of Work and Organisational Psychology, experienced healthcare and leadership researcher	Team working, collective leadership, safety culture, staff engagement, organisational innovation
External Advisor 2	Founder of the Collective Leadership Institute, systems scientist, consultant, strategic advisor in complex multi-stakeholder setting	Collective leadership, transformation, scaling-up collective stewardship skills, collaboration, sustainability.

## Abbreviations

CMOC: Context-mechanism-outcome configuration
IPT: Initial programme theory
RAMESES: Realist And MEta-narrative Evidence Syntheses: Evolving Standards
NHS: National Health Service (UK)
PCWs: Personal Care Workers
